# Supplementary figures and images for: The Gr64 cluster of gustatory receptors promotes survival and proteostasis of epithelial cells in Drosophila
Source: PLoS Biol. 2022 Jul 21;20(7):e3001710. doi: 10.1371/journal.pbio.3001710 (PMC9302837; doi:10.1371/journal.pbio.3001710)

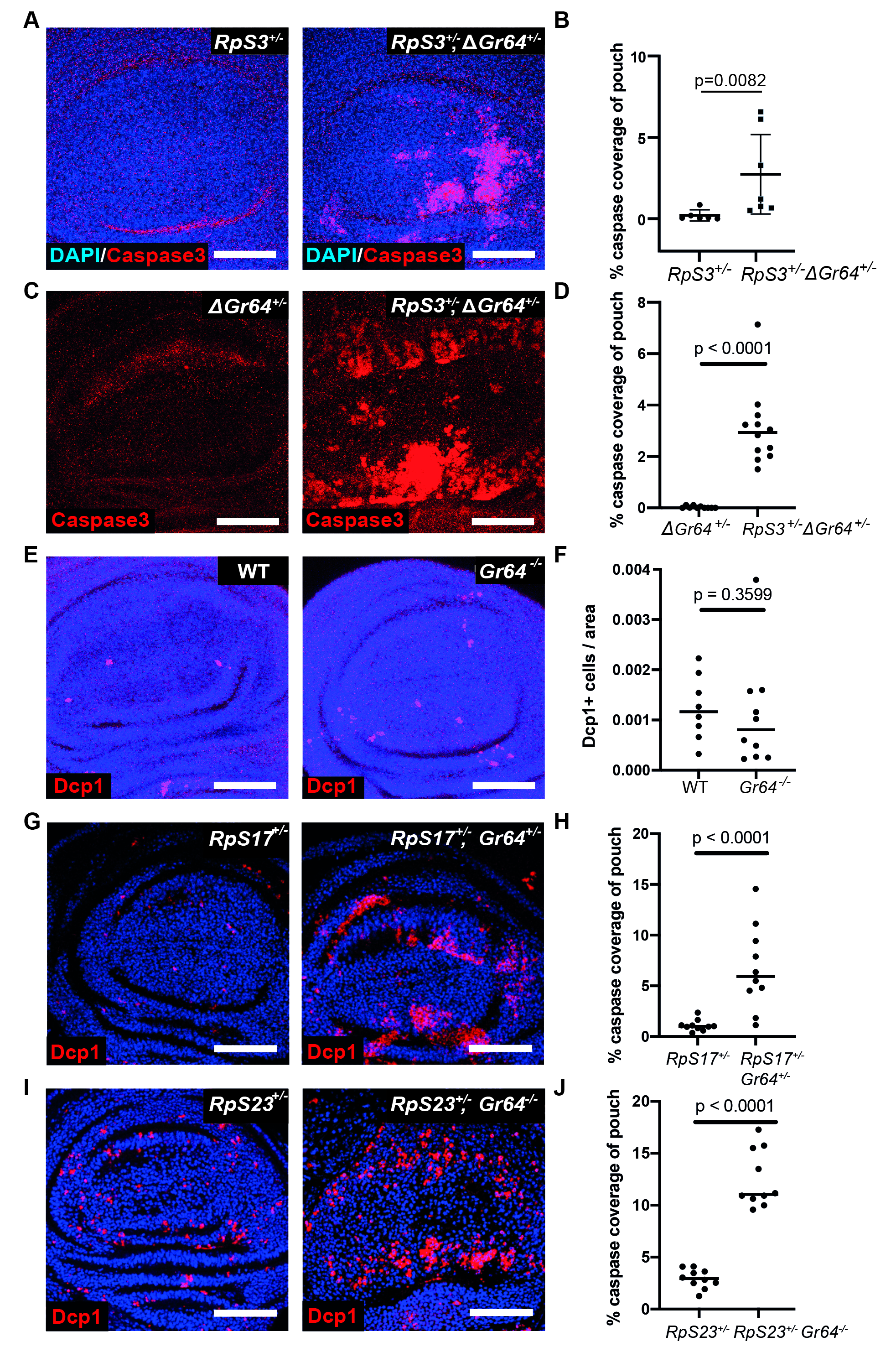

Supplement: S1 Fig — Further characterisation of the Gr64 and RpS3 interaction using additional Minute and Gr64 mutants. (A) Wing discs heterozygous mutant for RpS3 without (left panel) or with (right panel) a heterozygous deficiency in the Gr64 locus (ΔGr64) and assessed for cell death with a staining for cleaved-caspase3 (red), along with quantification in (B) (nRpS3 = 6, nRpS3,ΔGr64 = 7, 2-sided Mann–Whitney U test). (C) Wing discs heterozygous for a deficiency spanning the Gr64 locus (ΔGr64) in a wild type (left panel) or RpS3+/- background (right panel) and assessed for cell death with a staining for cleaved-caspase3 (red), along with quantification in (D) (n ΔGr64 = 10, nRpS3,ΔGr64 = 11, 2-sided Mann–Whitney U test). (E) Wild type (left panel) or Gr64 homozygous null (right panel) wing discs assessed for cell death via a staining for cleaved-Dcp1 (red), along with quantification in (F) (nwt = 8, nGr64 = 10, 2-sided Mann–Whitney U test). (G) Wing discs heterozygous for RpS17 (left panel) or heterozygous for both RpS17 and Gr64 (right panel) assessed for cell death with a staining for cleaved-Dcp1 (red), along with quantification in (H) (nRps17 = 10, nRps17,Gr64 = 10, 2-sided Mann–Whitney U test). (I) Wing discs heterozygous for RpS23 (left panel) or both heterozygous for RpS23 and homozygous null for Gr64 (Gr64af) (right panel) assessed for cell death with a staining for cleaved-Dcp1 (red), along with quantification in (J) (nRps23 = 10, nRps23,Gr64 = 10, 2-sided Mann–Whitney U test). Horizontal lines indicate the mean in S1B, S1D, S1F and the median in S1H and S1J. Numerical data can be found in the “S1 Fig” sheet of S1 Data. Gr64, Gustatory Receptor 64; Rp, ribosome protein. (TIF) [file pbio.3001710.s001.tif]

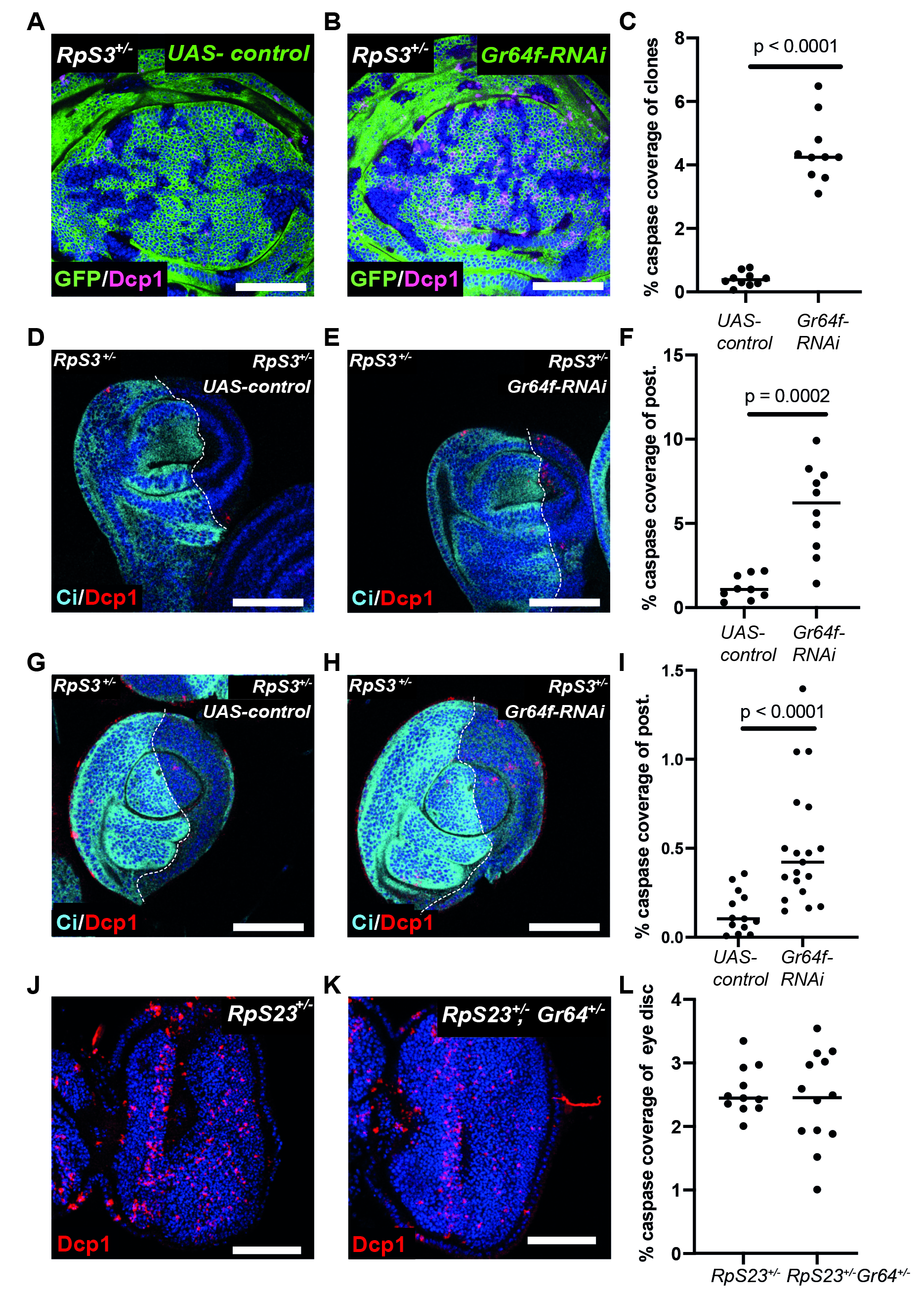

Supplement: S2 Fig — Characterisation of the Rps3 and Gr64 interaction in other imaginal discs. (A–C) Representative images of tubulin flp-out clones (green) in an RpS3 heterozygous background expressing a blank UAS control (A) or Gr64f-RNAi (B), assessed for cell death with a staining for cleaved-Dcp1 (magenta) along with quantification in (C) (nUAS-control = 10, nGr64f-RNAi = 10, 2-sided Mann–Whitney U test). (D–F) RpS3+/- haltere discs expressing a blank UAS control (D) or Gr64f-RNAi (E) in the posterior compartment with hh-Gal4, stained with anti-Ci (cyan) to label the anterior compartment, and assessed for cell death with a staining for cleaved-Dcp1 (red) along with quantification in (F) (nUAS-control = 9, nGr64f-RNAi = 10, 2-sided Wilcoxon signed rank test). (G–I) RpS3+/- leg discs expressing a blank UAS control (G) or Gr64f-RNAi (H) in the posterior compartment with hh-Gal4, stained with anti-Ci (cyan) to identify the anterior compartment, and assessed for cell death with a staining for cleaved-Dcp1 (red) along with quantification in (I) (nUAS-control = 13, nGr64f-RNAi = 19, 2-sided Wilcoxon signed rank test). (J, K) Eye discs heterozygous for RpS23 (J) or eye discs heterozygous for both RpS23 and Gr64 (K) assessed for cell death with a staining for cleaved-Dcp1 (red) along with quantification in (L) (nRps23 = 11, nRps23,Gr64 = 13, 2-sided Mann–Whitney U test). Horizontal lines indicate the median in all graphs. Numerical data can be found in the “S2 Fig” sheet of S1 Data. Gr64, Gustatory Receptor 64; RNAi, RNA interference; Rp, ribosome protein. (TIF) [file pbio.3001710.s002.tif]

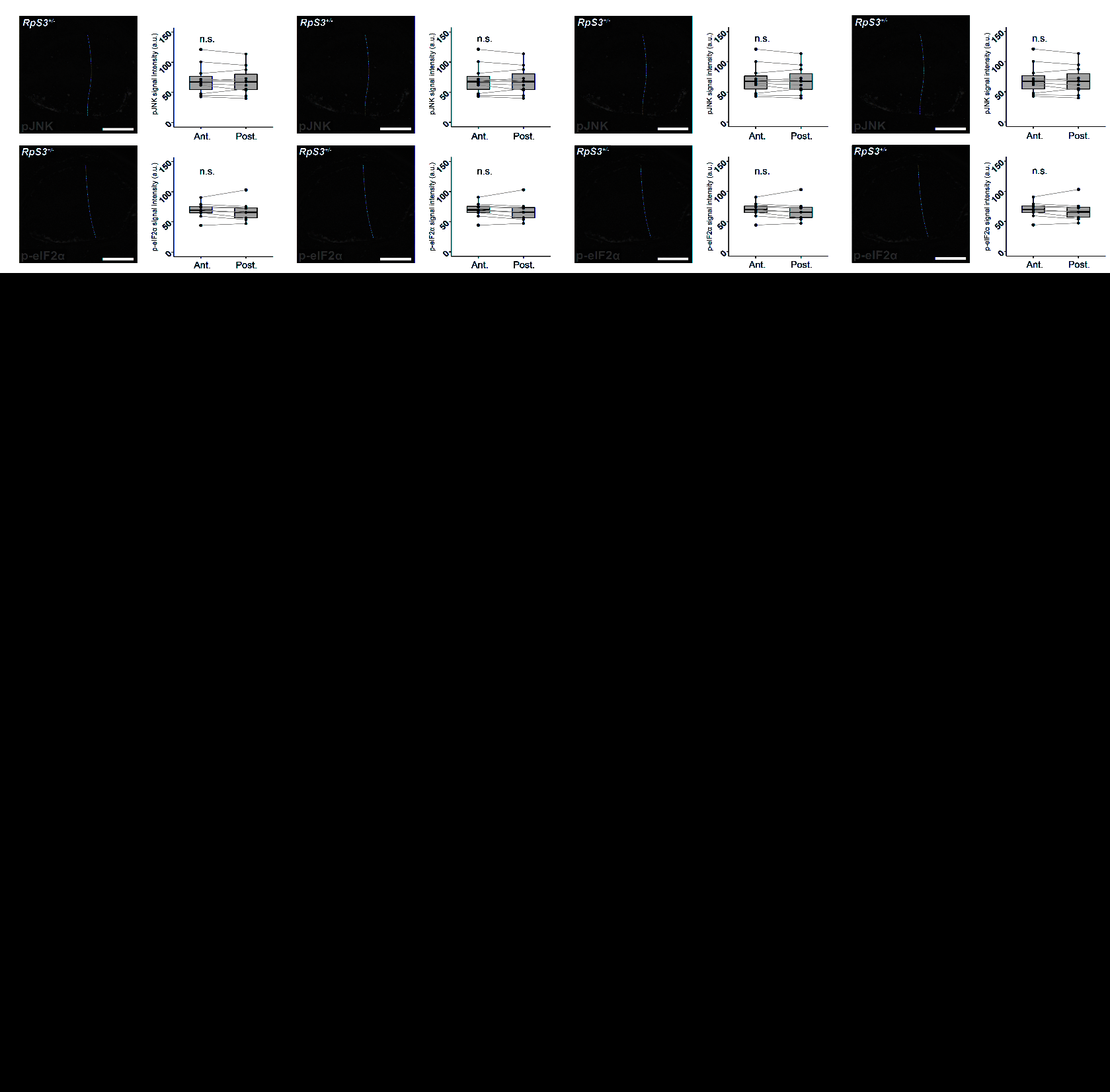

Supplement: S4 Fig — Negative (no RNAi) controls. (A) RpS3+/- wing discs carrying the enGal4 driver but expressing no RNAi construct in the posterior compartment and stained for phosphorylated JNK (green) with quantification in (B) (n = 11, 2-sided paired t test). (C) RpS3+/- wing discs carrying the enGal4 driver but expressing no RNAi in the posterior compartment and stained for phosphorylated eIF2α (green) along with quantification in (D) (n = 9, 2-sided paired t test). Numerical data can be found in the “S4 Fig” sheet of S1 Data. RNAi, RNA interference. (TIF) [file pbio.3001710.s004.tif]
