# Supplementary material for: The Gr64 cluster of gustatory receptors promotes survival and proteostasis of epithelial cells in Drosophila
Source: PLoS Biol. 2022 Jul 21;20(7):e3001710. doi: 10.1371/journal.pbio.3001710 (PMC9302837; doi:10.1371/journal.pbio.3001710)
Supplement: Attachment — Submitted filename: Baumgartner et al, rebuttal .pdf [file pbio.3001710.s010.pdf]

We thank the reviewers and the PLOS Biology editorial team for their feedback and suggestions on the manuscript. We are very thankful for the positive feedback of Reviewer 3. Special thanks also go to the editorial team and to the academic editor for judging that our revised manuscript meets the standards of a Discovery Report.

In the accompanying revised manuscript, we have addressed all final comments emerging from after the revision round. Specifics are detailed below.

CHANGES SUGGESTED BY THE EDITORIAL TEAM

In response to the requests of the editorial team, we have:

1) Provided all quantitations and their replicates in an excel file (S1_Data.xlsx supplemental file), wherein every sheet contains all quantitations for a given figure and its panels. This file is referred to in the material and methods, in the data availability statement and in every figure legend.
2) Added a data availability statement.
3) Added e reference to the model system (Drosophila melanogaster) in the abstract. It was already included in the title.
4) Toned down statements with respect to Gr64s acting exclusively cell autonomously (lines 145-150).
5) Added a discussion on the limitations of this work and on future work directions (line 248-250)
6) Submitted a 30-40 blurb describing the main findings.

COMMENTS FROM THE ACADEMIC EDITOR

1) In the tables in Figure 1A, the authors indicate "log2FC" values, which were taken from their previous study (Kucinski 2017). But digging into that paper (specifically, Supp Data 1) reveals the value are actually "log FC".

Original values in Kucinski et al., are log2FC, so we are correct in referring to them as log2FC in the current manuscript. It is true that they are referred to as logFC in Kucinski et al., however this is not wrong: while logFC usually refers to log10 it can also refer to log2.

2) In lines 112-113 the authors write "... they (Gr64 genes) do not appear to be expressed in wing discs (Flybase and Kucinski et al., 2017)". However the same dataset from (Kucinski 2017) indicate there is at least low-level expression of these genes. Therefore, the authors might want to nuance this statement (particularly as it seems relevant as to whether these genes might have a basal role in this tissue, or only become induced in the RpS3 mutant background).

We thank the academic editor for this comment. In Kucinski et al., we indeed report a very low level of counts (10-15) per isoform. This could just be noise, as RNA Seq can detect spurious reads or indeed it could indicate a very low expression level, as indicated by the academic editor. We have

therefore modified our statements to account for this. Instead of describing Gr64s as being strictly expressed in neuronal cells, we now state instead that their expression is “normally observed in neuronal cells” and that they become “upregulated” in epithelial cells in *Rp/+* mutant conditions (line 89). We have also replaced “not expressed” with “minimally expressed” (line 120).

REVIEWER COMMENTS

Reviewer #1: Dirk Bohmann (note this reviewer has signed his review)
Review of PBIOLOGY-D-21-02398R2

Baumgartner et al.

This paper describes a previously unrecognized phenotype of Drosophila mutants that are deficient for a certain group of taste receptors. Six Gr64 receptors are expressed from a polycistronic mRNA. Flies that are heterozygous for mutations in one of more of these 6 clustered genes display increased levels of oxidative stress and cell death in tissues that are also minute, i.e. heterozygous for ribosomal protein (rp) gene loss of function mutations. This combined rp+/-, Gr64+/- phenotype is observed regardless of whether the double heterozygous genotype is shared by all cells in the organism or whether the mutants are in a competitive situation next to wild type cells, in which case cell competition is expected.

All three reviewers of the first submission of this manuscript agree that the phenomenon described in is interesting and unexpected. It may give rise to new lines of research that may help to better understand the intriguing biology of minute mutants.

We thank Reviewer 2 for recognising that our observations on the function of Gr64 in epithelial cell proteostasis are interesting and unexpected.

The major concern that reviewer 2 and I pointed out was that the paper describes in interesting observation but does not go very far in providing insight into the functional basis for the unexpected interplay between smell receptors and minute mutants. In the revised version one experiment is added which indicates that in rp/+ imaginal discs cells in which Gr64 expression is knocked down calcium signaling is reduced. Demonstration of a connection between this effect and the observed phenotype cell stress phenotype would require addional experiments. At this stage such a connection is a matter of speculation. So, the question of novelty and general interest remains.

We disagree with this view. In this manuscript we provide a thorough initial characterization of the functional role of GR64 taste receptors in Minute cells. We go beyond showing that Gr64 receptors are required for rp/+ cell survival and demonstrate that GR64 activity is necessary to maintain proteotoxic stress in check: autophagy and proteasome functions are improved, proteotoxic and oxidative stress and protein aggregates are reduced by GR64 activity. Conversely, we show that translation is virtually unaffected. Altogether, these data allow us to demonstrate a novel proteostasis promoting role for Gr64 receptors. Our newest data, included in the revision, further suggest that this is likely mediated by changes in calcium signalling. Additional experiments to address how Gr64 promotes proteotoxic stress of course could be envisaged, but they are neither necessary for the scope of a Discovery Report, nor easy to carry out, given the pleiotropic nature of calcium signalling and of sugar manipulations that could affect Gr64 signalling. Overall, we believe

our data constitute a well-rounded report of the discovery of a non-neuronal function of taste receptors. In the words of reviewer 3: "It presents a significant discovery that will be interesting to a wide range of scientists."

The specific issues that I raised in my previous review included:

It is difficult to interpret the data presented in figure 2 where individual insertion mutants in the each of the six Gr64 receptors are tested for their effect on rp/+ dependent cell death. Reviewer 2 expressed a similar concern. The authors point out, correctly, that it is difficult to know whether mutations in the coding regions of one of the six genes would only affect the expression that particular gene product or also that of some or all of its neighbors. In the end I am still confused about how to interpret this result, beyond: these are six more independent alleles that can be presumed to affect Gr64 function in a somewhat undefined way that support the conclusion of the previous picture.

When we carried out this mini-screen to look at the role of all individual Gr64 isoforms in Minute competition, we could not envisage this result. First, it was not a given that we should have seen a worsening of cell competition, as pathways that affect Minute cells autonomously do not necessarily have to also affect cell competition. Second, we thought we might identify specific subunits with a stronger phenotype. The outcome is instead showing a similar effect for all subunits, with the exception perhaps of Gr64a, which appears to give the mildest effect. As discussed in the manuscript and mentioned by the reviewer above, this is due most likely to the fact that the polycistronic nature of the gene may cause individual gene isoform deletions to affect expression of neighbouring isoforms. While the data in Figure 2 present the above limitations in terms of interpretation, they do not simply "support the conclusion of the previous picture"; they provide unequivocal proof (as shown with 6 distinct alleles) that Gr64 removal also worsens the competitive nature of Minute cells.

I also wondered how clear it is that the effect of the rp+/-, Gr64+/- genotype is cell autonomous. There is clearly more cell death in the tissue regions in which Gr64 expression is reduced. But there is also some in adjacent areas. The newly added experiments looking at haltere and leg imaginal discs do not clarify this question. The conclusion of cell autonomy should be qualified.

We have added a sentence in the manuscript discussing the possible causes of the modest increase in cell death in the adjacent tissue, either stemming from some contributing systemic effect, or from apoptosis-induced apoptosis (line 145-147).

The other issues I pointed out were mostly suggestions to improve the paper.

Reviewer 2 has some additional technical comments, several of which were satisfactorily addressed:

* Test minute mutations other than Rps3
This is satisfactorily addressed by showing experiments on RpS17 and Rps23 (revised Fig S1)

* Test the interaction of minutes with a Gr64 homozygous loss of function genotype.
This was done, in an experiment with Rps23 (revised Fig S1). The presented results are consistent with the author's model.

*	Do a rescue experiment, where expression Gr64 receptors would revert the mutant phenotype.
This was done using a UAS construct that has enough expression without a Gal4 driver. The presented results support the authors conclusion and are responsive to reviewer 2's query.

We thank Reviewer 1 for recognising that our revision work has satisfactorily addressed the above requests made by Reviewer 2. These additional experiments not only provide compelling proof that the phenotypes observed are indeed specific to Gr64, they also widen the scope of our initial observations. They demonstrate that the dependence on Gr64 by rp+/- cells can be seen in other imaginal discs and that the interaction with Gr64 is not specific to RpS3 but can be observed in at least two other rp+/- mutations (RpS17 and RpS23).

In conclusion

The revision provides additional lines of experimental evidence in support of the proposed role of Gr64 receptors in the minute phenotype and cell stress responses. It does however not offer more conclusive functional insight into the mechanism underlying this phenomenon.

We have rebutted above this comment on the functional insight into the mechanism of Gr64 function.

Reviewer #3: I was asked to review this revised manuscript (The Gr64 cluster of gustatory receptors promotes survival and proteostasis of epithelial cells in Drosophila) for PLoS Biology after reviewing the original version for eLife. I was Reviewer 3 in the eLife reviews and wrote generally positive comments with only minor suggestions for revision. I was impressed by the original manuscript and see that the revisions in this version have only strengthened the presentation. In my opinion, you have adequately addressed the concerns presented by the eLife reviewers. You have certainly addressed the relatively minor concerns that I had.

To me, this manuscript fulfills all the requirements for publication as a Discovery Article. It presents a significant discovery that will be interesting to a wide range of scientists. It will be particularly interesting to researchers interested in ribosomopathies and cellular stress. The results are novel and they are presented and analyzed with appropriate rigor and clarity. The manuscript is well written and pleasant to read. I see no need for additional experiments or significant revisions.

I am personally intrigued by this line of research. I think the connection you have made between ribosome dysfunction and gustatory receptor signaling is quite interesting. I am impressed that you followed up on your preliminary observations regarding the Gr64 genes in Minute mutants and found a new cellular role for this receptor gene family.

We thank Reviewer 3 for supporting publication of our story as a Discovery Article and for continuing to praise our manuscript, the quality of the experiments therein and especially for recognising the the value of our discoveries for a wide range of scientists.
