# Supplementary material for: The Gr64 cluster of gustatory receptors promotes survival and proteostasis of epithelial cells in Drosophila
Source: PLoS Biol. 2022 Jul 21;20(7):e3001710. doi: 10.1371/journal.pbio.3001710 (PMC9302837; doi:10.1371/journal.pbio.3001710)
Supplement: Attachment — Submitted filename: Baumgartner et al, rebuttal.pdf [file pbio.3001710.s011.pdf]

We thank the academic editor and the PLOS Biology editorial team for their feedback and suggestions on the manuscript. In the accompanying revised manuscript, we have addressed the last few comments from the Academic Editor as follows:

COMMENTS FROM THE ACADEMIC EDITOR

1) Regarding my first comment on the previous version about the values in Figure 1A: I was mistaken in stating the "values are actually "log FC"". I meant to write in the Kucinski 2017 study Supp Table 1, the values are "Fold change" (i.e. neither log10 nor log2). The authors should double check this and correct.

Our biggest thank you for spotting this mistake. It is indeed the case that the data in Kucinski et. al, are fold changes and indeed we should have referred to them in those terms. We have several versions of those data in the lab , some of which are in Log2FC and this has generated the mistake. Many thanks for insisting on this point. We have now changed it to Fold changes in Figure 1.

2) The phenomenon of calcium flashes (Figure 4J) is intriguing as a potential physiological link between Gr64s and downstream cellular events, but as such flashes have not been described in the literature before - as far as I can see - it would be good to provide some context as to what the authors think they represent (from the video they seem to occur somewhat randomly in different cells/groups of cells in the disc). In addition, could the authors confirm that the small, but significant A-P asymmetry in frequency observed is really due to the Gr64 RNAi in the P compartment and not reflecting an inherent difference in calcium flash frequency in these two different regions of the disc (i.e. without any Gr64 RNAi)? If they do not have such data, they should at least mention this caveat.

Calcium flashes have been observed and characterised in wing disc epithelia and other fly epithelia before (*Balaji et al., 2017; Calcium spikes, waves and oscillations in a large, patterned epithelial tissue*). Although their role is not fully known, they are understood to play a role in patterning. Importantly, characterisation of calcium flashes has not revealed regional differences between And P compartments in wing discs. We believe therefore that our conclusion that GR64 RNAi is responsible for changes in calcium frequency between A and P compartments to be justified. We have included a reference to the above work in the updated manuscript.
