# Supplementary material for: The Gr64 cluster of gustatory receptors promotes survival and proteostasis of epithelial cells in Drosophila
Source: PLoS Biol. 2022 Jul 21;20(7):e3001710. doi: 10.1371/journal.pbio.3001710 (PMC9302837; doi:10.1371/journal.pbio.3001710)
Supplement: Attachment — Submitted filename: Gr64 revision plan.docx [file pbio.3001710.s007.docx]

Dear Eugenia,

Thank you for submitting the paper "Ribosome protein mutant cells rely on the GR64 cluster of gustatory receptors for survival and proteostasis in Drosophila" for consideration by eLife. Your article has been reviewed by 3 peer reviewers, and the evaluation has been overseen by a Reviewing Editor and a Senior Editor. The reviewers have opted to remain anonymous.

Comments to the Authors:

We are sorry to say that, after consultation with the reviewers, we have decided that this work will not be considered further for publication by eLife. The consensus is that the primary observation is interesting but that without any further understanding of this phenomenon, too many questions remain unanswered. In essence, the story is too preliminary for publication in eLife. However, we hope that the comments of the reviewers will help you to improve the manuscript for submission elsewhere.

----------

eLife's editorial process also produces an assessment by peers designed to be posted alongside a preprint for the benefit of readers.

Public Evaluation Summary:

This paper explores an unexpected cell response to stress caused by the abnormal protein synthesis that occurs when a protein component of ribosomes is present in insufficient amounts; namely, the increased expression of gustatory receptors. It shows that the receptors function to reduce proteotoxic stress by upregulating stress pathways and proteasome and autophagy functions. This paper will certainly be interesting to scientists investigating ribosomopathies, but its larger impact may be in implicating a family of receptors in cell stress responses.


Reviewer #1 (Public Review):

This paper investigates mechanisms that modulate the phenotype of cells that are heterozygous for ribosomal protein genes (Rp/+). The previously described effect of the Rp/+ or minute genotype in completely heterozygous animals is delayed development and increased levels of proteostasis stress, which correlates with decreased autophagy and proteasome activity. In a clonal situation where Rp/+ cells are juxtaposed to wild type cells, they get eliminated by cell competition and apoptosis.

Based on results of transcriptomic analyses, the authors found that six clustered tasteGr64 receptor genes are more highly expressed in Rp/+ than in wild type cells. In loss of function experiments they show that heterozygous loss of the Gr64 cluster and even loss of heterozygosity for any one of these six genes or RNAi mediated knock down aggravates the stress response in Rp/+ cells and promotes apoptosis when these cells are in competition with neighboring wild type cells. Reporter experiments and immunostaining indicates that the Gr64, Rp double heterozygous cells experience higher levels of oxidative and unfolded protein stress. Heterozygosity or even complete loss Gr64 genes does not cause apoptosis of elevated stress markers in a wild type background.

This paper leaves a lot of questions open: What is the explanation of individual loss of function alleles for all six Gr64 receptors having the same dominant phenotype in an Rp/+ background? What is the molecular function of these receptors that modulates to the minute phenotype? Are any ligands involved? If so, which?

Our experiments show that most allelic mutations at GR64 loci worsen the Minute cells state. As the GR64 locus is polycistronic, it is likely that each of these mutations affects multiple isoforms, as also suggested by Reviewer 2 below. We think that the strength of these data is in the fact that multiple alleles share the same phenotype making it very robust and reproducible. Given the complexity of the genes locus however this may not necessarily indicate that loss of all individual receptors has a dominant effect and we have been careful not to draw that conclusion in the manuscript.

Regarding the molecular function of these receptors that modulate the minute phenotype: We are now able to answer this question. We have generated new data since submission of this manuscript that indicates that GR64s affect calcium signalling in Minute cells, providing a molecular mechanism for the observed phenotypes. Together with this new data are manuscript shows that GR64 genes are upregulated in ribosome mutants and improve the viability of these cells by activating protein catabolism pathways, through modulation of intracellular calcium levels. We will add those data to the revised manuscript.

Establishing whether ligands are involved and which ones is not feasible in a simple way. Several aspects of Minute cell competition and of the Minute cell state are affected by the diet and so any changes in phenotype observed upon manipulations of feeding conditions would be difficult to attribute unequivocally to their role in Gr64 signalling. In addition, we note that this manuscript was submitted to eLife and is now submitted to Plos Biology as a short report. As a short report, our manuscript does not need to provide a full mechanistic characterisation of GR64 function in the context of ribosome gene loss. Our manuscript identifies for the first time a role in non-neuronal cells for GR64, a and a new function -regulation of proteostasis – in a new cellular pathology (ribosome- induced proteotoxic stress). We believe this constitute ample and important novelty at a level expected for a short report and that identifying the GR64 ligands with a function in cell competition is not only impracticable but also beyond the scope of this short manuscript.

Reviewer #1 (Recommendations for the authors):

Figure 1 and supplemental Fig 1: Why do you show the experiment with the larger deficiency that requires complicated complementation with rescue constructs when you have a precise CRISPR deletion?

Thanks for pointing that out. Indeed, we have done both the experiments with the deficiency and with the Crispr deletion and got qualitatively the same results. Showing that independent alleles give the same result is an important confirmation that this is not due to passenger mutations/genetic background. For these reasons both experiments were included in the manuscript with the Crispr mutant experiments shown as supplementary data. We agree that, as this is a cleaner experiment, we should swap those around and show the large deletion data as Supplementary instead.

Figure 2a: why is no apoptosis detectable at the borders between WT and RpS3/+ clones?

There are only two small apoptotic bodies in that wing disc, which is lower than there normally should be. We recognise that the image is misleading, and we will replace it with a different representative image accordingly.

Some minor issues that should be addressed in the paper:

Line 108: "ΔGr64/ΔGr64 flies present with no known phenotypes other than a deficient gustatory responses" should be "than a deficient gustatory response" or "than deficient gustatory responses")

Thank you for picking that.

Line 129: "heterozygousity" should be "heterozygosity"

Thank you for picking that typo.

It would be helpful if the figures and supplemental figure were identified by the numbers that refer to them in the text.

Absolutely -We will do that.

Label the genotypes in the figures better, which drivers are used etc.

We will do that. In addition all detailed genotypes of all experiments are already provided in a supplementary table.

Fig 1E,F. It is not clear why the paper suggests that knockdown in Hh-expression domain which span most of a parasegment shows a cell-autonomous requirement for Gr64. In fact, there are Caspase positive cells outside the Hh-expression domain. Clonal knock down for example by MARCM and precise analysis at the clonal boundaries would be the accepted method to test cell autonomy.

While there are some caspase positive cells in the Anterior compartment careful quantification, as shown in Fig. 1G shows between 5 and 20 times more cell death in the posterior compartment where Gr64 is silenced than in the control Anterior compartment. That indicates that by and large the effect of Gr64 is cell-autonomous. However we are happy to provide further demonstration of this by carrying out the MARCM experiments indicated by the reviewer.

We also not that the small increase in cell death in the Anterior compartment could be also due to an unrelated phenomenon known to take place in wing discs when one compartment has massive levels of cell death: apoptosis-induced apoptosis.

Reviewer #2 (Public Review):

Baumgartner et al. follow up a curious observation they made previously (Kucinski et al. 2017) , in which they noticed that Drosophila larvae heterozygous for the ribosomal protein Rsp3 show upregulation of sugar taste receptor genes of the Gr64 subfamily (Gr64a-Gr64f). They test a functional involvement of these receptors by creating double heterozygous Rsp3+/-, Gr64a-f+/-, larvae and analyzing imaginal discs for evidence of increased proteotoxic stress/apoptosis compared to Rsp3+/- imaginal discs and find the former have indeed an increase in expression of caspase3 and DCP1, stress/cell death markers. In a slightly modified experiment (Figure 2), they investigate whether this phenomenon is also seen in discs in which winner (Rsp3+/+) and loser cells Rsp3+/-) are placed adjacent to each other, finding that small groups of DCP1 expressing cells are indeed more frequent at the interface of winner/loser cells in heterozygous Gr64 discs, compared to Gr64+/+ discs. They develop a more nuanced assay, in which they downregulate Gr64s via RNAi, and by expressing this construct in the posterior part of the disc. In this assay, they observe a comparatively milder effect on apoptosis, assayed by several different markers. Employing this more sensitive assay, they explore whether Gr64a contributes to protein aggregation using two reporters that represent proteolytic pathways and poly-ubiquitinylated aggregates.


Reviewer #2 (Recommendations for the authors):

Strengths:

1) A non-canonical role for taste receptors in ribosome biogenesis is a very interesting finding. While no experiments to address the mechanism are presented, the observation presented here open up new lines of investigations.

Our manuscript does identify a mechanism for the role of GR64 in ribosome mutants. We show that GR64 activity is necessary to maintain proteotoxic stress in check: autophagy and proteasome functions are improved and proteotoxic stress and protein aggregates are reduced by GR64 activity. How proteotoxic stress is regulated by GR64 remained to be determined. The likeliest explanation, given the role of Gr64 in calcium signalling in neurons, was that this is mediated by modulation of calcium signalling. Therefore, to address this comment, we have done additional experiments and tested this hypothesis. Live imaging of intracellular calcium levels shows that indeed GR64 modify intracellular calcium signalling in ribosome mutant epithelial cells. This indicates that GR64 improves proteostasis via modulation of calcium signalling. We will add these new data to the revised manuscript.

2) The experiments are well-designed, and appropriate controls are included.

Thank you

3) The paper is well-written and easy to digest.

Thank you

Weaknesses:

1) The authors should extend the analysis further, including phenotype assessment of the minute phenotype in hemi- and homozygous Gr64 mutant that are Rsp3+/-. Likewise, why did they never use homozygous Gr64 mutants in any of their experiments? The effects on the observed phenotype would be expected to be even stronger.

These are valid objections, and assessing, for instance, autophagy phenotypes in *RpS3^+/-^*, *Gr64^+/-^* wing discs is doable and would be informative. In the original manuscript, we preferred the Gr64-RNAi condition for asking these questions, as it allowed us to assess proteotoxic stress phenotypes in the absence of broad cell death, which is observed in heterozygous conditions. As pre reviewer request, we will recapitulate the findings seen in the milder RNAi condition in the mutant context, as confirmation.

In regards to the question as to why no *Gr64* homozygous mutant experiments were performed, this was due to both the sickliness of the larvae and the position of the gene on the chromosome. As both *RpS3* and *Gr64* are on the third chromosome, a recombinant is needed. We however struggled with generating such a recombinant, as the necessary cross was sickly. We tried multiple recombinant strategies, but ultimately discontinued this plan due to the technical challenges.

However, since we show in the manuscript that the heterozygous phenotypes of multiple alleles give consistently a pronounced phenotype, we feel that we have multiple independent confirmations of the role Gr64 is playing and that the homozygous condition would not be particularly informative.

We will nevertheless attempt one more strategy and test whether we can obtain larvae that are Gr64 homozygous mutant and heterozygous for a different Minute gene.

2) Interpretation of the data in Figure 2 is difficult. These mutations are mostly gene knock-ins into a locus that is transcribed from a polycistronic message, so any of these mutations can affect levels of expression of the entire locus. While these knock-ins mostly the purpose to reveal cellular expression but using them as gene knock-outs is fraught with many potential caveats.

The reviewer correctly points out that knocking out one gene may influence the expression of adjacent genes (this has in fact been shown in the Gr64 context itself). We would counter, however, that this knockout strategy has been used successfully in the prior literature to identify ligands specific to distinct Gr64 subtypes, indicating that to a good approximation they primarily affect the specific isoform targeted by the knock-in. However we fundamentally agree with the caveats raised and indeed we were very careful not to draw strong conclusions or make specific claims as to which Gr64 subtypes are relevant to competition. We will therefore address this comment by clarifying our purpose in conducting these experiments, which was to show an involvement of the Gr64 locus in competing Minute losers. We will supplement the text with a discussion on the literature showing knock-on effects of interfering with individual genes in a complex locus.

3) There are no "rescue" experiments in this paper. It appears that it would be very valuable to see if the observed phenotypes (Capsase3, DCP1 expression) incurred by heterozygosity of the Gr64 locus can be rescued by expression of individual G64 genes.

We have previously attempted some rescuing experiments using UAS-Gr64 constructs. Expressing Gr64 genes with UAS in *RpS3* cells has proven highly toxic. However, these results are not physiological, as overexpression of Gr64s in this way does not mimic the low-level expression seen in the RNA-seq. We will explore this further and use weaker drivers to see if we can achieve optimal expression, such as gal80ts modulated expression.

4) The authors' previous data indicate that upregulation of Gr64 genes in Rsp3+/- (and other heterozygous mutants of genes involved in ribosome biogenesis) larvae might be a counter measure by increasing intracellular Ca2+. They do not provide an opinion whether this would be a ligand dependent or independent process. A simple test for that would be a rescue type of experiments with other Gr genes (see point 4) not related to sugar receptors)

As mentioned above, in data we had not included in the manuscript we have observed that UAS-driven expression of GR64 receptors is toxic to Rsp3+/- cells, probably because this reaches unphysiologically high levels of expression. On this basis it is unlikely that UAS-driven expression of a different Gr gene could yield a rescue. As an alternative we propose to examine directly how GR64 impacts intracellular calcium levels in RpS3 cells using GCaMP reporters. This will allow us to monitor both steady state calcium levels and calcium flushes and determine how they are affected by GR64 genes in RpS3+/- cells.

Minor points:
1) nomenclature for Gr64 mutants is not accurate and confusing (Gr64a-GAL4, Gr64b-LexA, etc). They should refer to the original nomenclature used by Fujii et al. (Gr64aGAL4 etc)

Thanks for pointing this out. We will.

2) The authors initiate the investigation on a possible role of Gr64 genes based on differential expression (i.e. upregulation) of these genes in Rsp3+/- and other mutants. This was a very restricted analysis (wing imaginal disc). Is this phenomenon observed in other discs/tissues that form adult structures?

We will investigate this directly by quantitative RTPCR of leg and antenna discs as well.

Reviewer #3 (Public Review):

I found this paper quite satisfying, because it is an excellent example of "following the science" to discover something new and totally unexpected about the way cells work. I thought the story was compelling, the presentation was clear and well written, and that the impact will be significant.

The authors followed up on observations made in a previous paper (Kucinski et al., 2017), where they compared gene expression in wild type flies versus flies heterozygous for a mutation in a ribosomal protein gene or another gene, mahjong, associated with proteotoxic stress. The analysis resulted in a long list of differentially expressed genes, some of which they explored in that paper. Here, they explore the reasons for the unusual upregulation of a family of gustatory receptors. Rather than representing a meaningless case of gene misregulation, they found that increased expression of these receptors is an adaptive response to proteotoxic stress. In this way, they implicated the receptors in regulating pathways that no one had suspected them of regulating.

I have no criticisms of the authors' choice of experiments, the methods they used, their interpretations or their presentation. The major strength of this paper is the soundness of the methods and results. It is an exceptionally "clean" and well-constructed report.

For me, the significance of the work relates to the long history of ribosomal protein gene haploinsufficiency in Drosophila and the increasing importance of Drosophila to understanding cellular responses to disrupted protein synthesis. A relatively short time ago, explanations for the Minute syndrome-the phenotypes seen in Drosophila when one copy of a ribosomal protein gene is eliminated-were speculative. The syndrome was considered an esoteric "fly thing". Over the past decade or so, the phenotypes have been tied to an assortment of cell stresses. A major challenge now is sorting through all the ways ribosome dysfunction affects cellular pathways. It is a complicated situation, but it mirrors cellular defects in human ribosomopathies and other diseases affecting protein synthesis. This paper is an excellent example of the relevance, importance and productivity of such studies: a new cellular pathway linking gustatory receptors to cellular stress responses has now been discovered from its contribution to the Minute syndrome.

We would like to thank reviewer #3 for their kind words regarding the project.

Reviewer #3 (Recommendations for the authors):

After reading this paper, one is left with the impression that what you describe for reduce RpS3 dosage is universal to reduced dosage for all Rp genes. I would like to have seen some statement that interactions between RpS3 and the Gr64 genes might be RpS3 specific. Better yet, I would like to have seen at least some experimental evidence showing the same interactions with another Rp gene. While I understand why you think the effects are probably not RpS3 specific, I urge to be hesitant and modest in generalizing. I recommend including appropriate caveats in the text.

This is indeed a very important point, which we will address not simply by discussion but by addition of novel experiments which demonstrate that these interactions are not limited to RpS3. We will show that another ribosome genes mutant shows similar interactions and requirement of Gr64 genes for viability.

The text is very well written, but I had a hard time understanding the section on "flux" beginning with line 156. "Flux" is not a word that is universally understood. I had to go back to your previous paper to figure out what you were talking about and to understand the purposes of your ProteoFlux and ReFlux tools. For ease of reading, I strongly recommend that you use more familiar phrases such as "import and degradation", "elimination", etc. and provide better descriptions of the ProteoFlux and ReFlux tools.

Thanks for pointing this out. We will improve terminology and replace the term flux with more accessible terms for ease of reading.

Because an asterisk is used to denote an unknown allele, I wanted to know why you used RpS3[*] in the allele designation in the experiments associated with Figure 1, but I could find no description of the origin of the allele. From context, I suspect it came from Bloomington stock 5699. Please include a note about its origin somewhere (or, better yet, sequence it and the known alleles and identify it definitively).

Thanks for pointing this out. We will.

On line 66, you meant to use "proximate", not "proximal".

Thanks. We will correct accordingly.

You should use FlyBase nomenclature for the genetic elements in your experiments, especially transgenes, at least once so that readers will not have to guess the correspondences between your lab notations and the carefully curated FlyBase entries describing them. Shortened symbols are OK for the text, but standardized symbols should be included in the Materials and Methods. This should be done for the sake of accuracy and reproducibility and to show that you value the efforts of the people who work hard organizing and archiving genetic information for you. Along the same lines, you should acknowledge research resources such as FlyBase that have enabled your work by explicitly citing their grants.

Thanks for pointing this out. We will.
