# Supplementary material for: The Gr64 cluster of gustatory receptors promotes survival and proteostasis of epithelial cells in Drosophila
Source: PLoS Biol. 2022 Jul 21;20(7):e3001710. doi: 10.1371/journal.pbio.3001710 (PMC9302837; doi:10.1371/journal.pbio.3001710)
Supplement: Attachment — Submitted filename: Baumgartner et al, rebuttal 4.docx [file pbio.3001710.s009.docx]

We thank the reviewers and the PLOS Biology editorial team for their feedback and suggestions on the manuscript. In the accompanying revised manuscript, we have made our best effort to address their comments, and we believe that, as a result, the manuscript has improved greatly. In particular, newly added experiments:

1. Provide a demonstration that a UAS-GR64 transgene rescues the Gr64 mutant phenotype. This removes any doubt that the phenotype we had reported was not specific to the mutation. This was already unlikely as we had demonstrated this effect using two independent mutants and one RNAi line.
2. Show that GR64’s role in promoting proteostasis is observed across different ribosome mutants. In addition to our initial work with RpS3, in the revised manuscript we show that mutations in RpS17 and RpS23 are also sensitive to Gr64 gene dosage.
3. Show that Gr64 survival role can be seen in several types of imaginal discs, including haltere and leg discs.
4. Show that Gr64 inhibition affects calcium signalling in RpS3 cells, by imaging a reporter of intracellular calcium. This provides support to our hypothesis that Gr64 promotes proteostasis via modulation of calcium signalling.

Reviewer #1 (Public Review):

This paper investigates mechanisms that modulate the phenotype of cells that are heterozygous for ribosomal protein genes (Rp/+). The previously described effect of the Rp/+ or minute genotype in completely heterozygous animals is delayed development and increased levels of proteostasis stress, which correlates with decreased autophagy and proteasome activity. In a clonal situation where Rp/+ cells are juxtaposed to wild type cells, they get eliminated by cell competition and apoptosis.

Based on results of transcriptomic analyses, the authors found that six clustered tasteGr64 receptor genes are more highly expressed in Rp/+ than in wild type cells. In loss of function experiments they show that heterozygous loss of the Gr64 cluster and even loss of heterozygosity for any one of these six genes or RNAi mediated knock down aggravates the stress response in Rp/+ cells and promotes apoptosis when these cells are in competition with neighboring wild type cells. Reporter experiments and immunostaining indicates that the Gr64, Rp double heterozygous cells experience higher levels of oxidative and unfolded protein stress. Heterozygosity or even complete loss Gr64 genes does not cause apoptosis of elevated stress markers in a wild type background.

This paper leaves a lot of questions open: What is the explanation of individual loss of function alleles for all six Gr64 receptors having the same dominant phenotype in an Rp/+ background? What is the molecular function of these receptors that modulates to the minute phenotype? Are any ligands involved? If so, which?

Our experiments show that most allelic mutations at GR64 loci worsen the Minute cells state. As the GR64 locus is polycistronic, it is likely that each of these mutations affects multiple isoforms, as also suggested by Reviewer 2 below. We think that the strength of these data is in the fact that multiple alleles share the same phenotype, making it very robust and reproducible. Given the complexity of the genes locus, however this may not necessarily indicate that loss of all individual receptors has a dominant effect and we have been careful not to draw that conclusion in the manuscript.

Regarding the molecular function of these receptors that modulate the minute phenotype, our revised manuscript suggests a possible explanation. We include data that indicates that GR64s affect calcium signalling in Minute cells, providing a possible molecular mechanism for the observed phenotypes. Indeed our manuscript shows that GR64 genes are upregulated in ribosome mutants and improve the viability of these cells by activating protein catabolism pathways. As calcium signalling is known to control proteostasis in a number of ways we suggest that Gr64 promote proteostasis through modulation of intracellular calcium levels.

Establishing whether ligands are involved and which ones is not feasible in a simple way. Several aspects of Minute cell competition and of the Minute cell state are affected by the diet and so any changes in phenotype observed upon manipulations of feeding conditions would be difficult to attribute unequivocally to their role in Gr64 signalling. In addition, we note that this manuscript is submitted as a report. In it we identify for the first time a role in non-neuronal cells for GR64, a and a new function -regulation of proteostasis – in a new cellular pathology (ribosome- induced proteotoxic stress). We believe this constitute ample and important novelty at a level expected for a report and that identifying the GR64 ligands with a function in cell competition is not only impracticable but also beyond the scope of this short manuscript.

Reviewer #1 (Recommendations for the authors):

Figure 1 and supplemental Fig 1: Why do you show the experiment with the larger deficiency that requires complicated complementation with rescue constructs when you have a precise CRISPR deletion?

Thanks for pointing that out. Indeed, we have done both the experiments with the deficiency and with the Crispr deletion and got qualitatively the same results. Showing that independent alleles give the same result is an important confirmation that this is not due to passenger mutations/genetic background. For these reasons both experiments were included in the manuscript with the Crispr mutant experiments shown as supplementary data. We agree that, as this is a cleaner experiment, we should swap those around and show the large deletion data as Supplementary instead. In the revised manuscript we have done that.

Figure 2a: why is no apoptosis detectable at the borders between WT and RpS3/+ clones?

There were two small apoptotic bodies visible in that specific wing disc section, which we agree is not a representative number. Several more wound have been found moving more basally in that wing disc, as this is where apoptotic bodies accumulate. We have now replaced that image with a representative one. We note in addition that the quantifications shown in H and I are done in 3D and span the entire thickness of the discs analysed.

Some minor issues that should be addressed in the paper:

Line 108: "ΔGr64/ΔGr64 flies present with no known phenotypes other than a deficient gustatory responses" should be "than a deficient gustatory response" or "than deficient gustatory responses")

Thank you for picking that.

Line 129: "heterozygousity" should be "heterozygosity"

Thank you for picking that typo.

It would be helpful if the figures and supplemental figure were identified by the numbers that refer to them in the text.

Absolutely -We have done that.

Label the genotypes in the figures better, which drivers are used etc.

We have added information on the drivers in the corresponding legends. In addition all detailed genotypes of all experiments are provided in a supplementary table.

Fig 1E,F. It is not clear why the paper suggests that knockdown in Hh-expression domain which span most of a parasegment shows a cell-autonomous requirement for Gr64. In fact, there are Caspase positive cells outside the Hh-expression domain. Clonal knock down for example by MARCM and precise analysis at the clonal boundaries would be the accepted method to test cell autonomy.

While there are some caspase positive cells in the Anterior compartment careful quantification, as shown in Fig. 1G, shows between 5 and 20 times more cell death in the posterior compartment where Gr64 is silenced than in the control Anterior compartment. That indicates that by and large the effect of Gr64 is cell-autonomous. The small increase in cell death in the Anterior compartment could be due to an unrelated phenomenon known to take place in wing discs when one compartment has massive levels of cell death: apoptosis-induced apoptosis.

However, to provide further demonstration we have included MARCM experiments, as indicated by the reviewer. These can be found in S2 Fig.

Reviewer #2 (Public Review):

Baumgartner et al. follow up a curious observation they made previously (Kucinski et al. 2017) , in which they noticed that Drosophila larvae heterozygous for the ribosomal protein Rsp3 show upregulation of sugar taste receptor genes of the Gr64 subfamily (Gr64a-Gr64f). They test a functional involvement of these receptors by creating double heterozygous Rsp3+/-, Gr64a-f+/-, larvae and analyzing imaginal discs for evidence of increased proteotoxic stress/apoptosis compared to Rsp3+/- imaginal discs and find the former have indeed an increase in expression of caspase3 and DCP1, stress/cell death markers. In a slightly modified experiment (Figure 2), they investigate whether this phenomenon is also seen in discs in which winner (Rsp3+/+) and loser cells Rsp3+/-) are placed adjacent to each other, finding that small groups of DCP1 expressing cells are indeed more frequent at the interface of winner/loser cells in heterozygous Gr64 discs, compared to Gr64+/+ discs. They develop a more nuanced assay, in which they downregulate Gr64s via RNAi, and by expressing this construct in the posterior part of the disc. In this assay, they observe a comparatively milder effect on apoptosis, assayed by several different markers. Employing this more sensitive assay, they explore whether Gr64a contributes to protein aggregation using two reporters that represent proteolytic pathways and poly-ubiquitinylated aggregates.


Reviewer #2 (Recommendations for the authors):

Strengths:

1) A non-canonical role for taste receptors in ribosome biogenesis is a very interesting finding. While no experiments to address the mechanism are presented, the observation presented here open up new lines of investigations.

Our manuscript provides an initial mechanism for the role of GR64 in cells with proteotoxic stress. We show that GR64 activity is necessary to maintain proteotoxic stress in check: autophagy and proteasome functions are improved and proteotoxic stress and protein aggregates are reduced by GR64 activity. How proteotoxic stress is regulated by GR64 remains to be determined. The likeliest explanation, given the role of Gr64 in calcium signalling in neurons, is that this is mediated by modulation of calcium signalling. Therefore, to address this comment, we have done additional experiments and tested this hypothesis. Live imaging of intracellular calcium levels shows that indeed GR64 modify intracellular calcium signalling in ribosome mutant epithelial cells (Fig. 4 and supplementary movie). This siggests that GR64 improves proteostasis via modulation of calcium signalling.

2) The experiments are well-designed, and appropriate controls are included.

Thank you

3) The paper is well-written and easy to digest.

Thank you

Weaknesses:

1) The authors should extend the analysis further, including phenotype assessment of the minute phenotype in hemi- and homozygous Gr64 mutant that are Rsp3+/-. Likewise, why did they never use homozygous Gr64 mutants in any of their experiments? The effects on the observed phenotype would be expected to be even stronger.

These are valid objections, and assessing, for instance, autophagy phenotypes in *RpS3^+/-^*, *Gr64^+/-^* wing discs would be informative. However, we preferred the Gr64-RNAi condition for asking these questions, as it allowed us to: 1) assess proteotoxic stress phenotypes in the absence of broad cell death, which is observed in heterozygous conditions; 2) availability of an internal control (the A compartment) to detect phenotypic changes in a sensitive manner.

In the revised manuscript we have extended our mutant analysis by showing that:

1) *GR64* mutants induce cells death in two other *Minute* mutant backgrounds (S1 Fig)
2) *GR64* mutants induce cell death also in leg and haltere discs, but not in eye discs (S2 Fig)
3) We further show that the *GR64^+/-^ RpS3^+/-^* mutant phenotype can be rescued by a *UAS-Gr64* transgene (Fig 1)

In regards to the question as to why no *Gr64* homozygous mutant experiments were performed, this was due to both the sickliness of the larvae and the position of the gene on the chromosome. As both *RpS3* and *Gr64* are on the third chromosome, a recombinant is needed. We however struggled with generating such a recombinant, as the necessary cross was sickly. We tried multiple recombinant strategies, but ultimately discontinued this plan due to the technical challenges. In the revision however we have been able to obtain *Gr64* homozygous mutants with a different *Minute* (*Rps23*) and show that *GR64^-/-^ RpS23^+/-^* discs also show substantial increase in cell death relative to *RpS23^+/-^* discs (S1 Fig).

2) Interpretation of the data in Figure 2 is difficult. These mutations are mostly gene knock-ins into a locus that is transcribed from a polycistronic message, so any of these mutations can affect levels of expression of the entire locus. While these knock-ins mostly the purpose to reveal cellular expression but using them as gene knock-outs is fraught with many potential caveats.

The reviewer correctly points out that knocking out one gene may influence the expression of adjacent genes (this has in fact been shown in the *Gr64* context itself). We would counter, however, that this knockout strategy has been used successfully in the prior literature to identify ligands specific to distinct Gr64 subtypes, indicating that to a good approximation they primarily affect the specific isoform targeted by the knock-in. However, we fundamentally agree with the caveats raised. We think that the strength of these data is in the fact that multiple alleles share the same phenotype, making it very robust and reproducible. Given the complexity of the gene locus, however this may not necessarily indicate that loss of all individual receptors has a dominant effect and we have been careful not to draw that conclusion in the manuscript.

.

3) There are no "rescue" experiments in this paper. It appears that it would be very valuable to see if the observed phenotypes (Capsase3, DCP1 expression) incurred by heterozygosity of the Gr64 locus can be rescued by expression of individual G64 genes.

We have previously attempted some rescuing experiments using UAS-Gr64 constructs. Expressing Gr64 genes with a Gal4 driven UAS in *RpS3^+/-^* cells has proven highly toxic (See e.g. new Fig 1D). This is not surprising, as overexpression of Gr64s in this way does not mimic the low-level expression seen in the RNA-seq data. We are pleased to report however that in the revision work we have successfully made use of the observation that a previously generated UAS-Gr64 construct shows rescuing effects even without a Gal4 driver (Slone et al., 2007). Our experiments show that indeed a UAS-Gr64 transgene rescues almost fully the lethality induced by Gr64^+/-^ in *RpS3^+/-^* cells.

4) The authors' previous data indicate that upregulation of Gr64 genes in Rsp3+/- (and other heterozygous mutants of genes involved in ribosome biogenesis) larvae might be a counter measure by increasing intracellular Ca2+. They do not provide an opinion whether this would be a ligand dependent or independent process. A simple test for that would be a rescue type of experiments with other Gr genes (see point 4) not related to sugar receptors)

As mentioned above, UAS-driven expression of Gr64 receptors is toxic to *Rsp3^+/-^* cells (new Fig 1D), probably because this reaches unphysiologically high levels of expression. On this basis it is unlikely that UAS-driven expression of a different Gr gene could yield a rescue. As an alternative to address the link between Gr64 and calcium signalling in *RpS3^+/-^* cells, we have examined how GR64 impacts intracellular calcium levels using GCaMP reporters. Our data indicate that Gr64 facilitates calcium signalling in *RpS3^+/-^* cells (Fig 4J), consistent with the hypothesis proposed by the reviewer.

Minor points:
1) nomenclature for Gr64 mutants is not accurate and confusing (Gr64a-GAL4, Gr64b-LexA, etc). They should refer to the original nomenclature used by Fujii et al. (Gr64aGAL4 etc)

Thanks for pointing this out. We will.

2) The authors initiate the investigation on a possible role of Gr64 genes based on differential expression (i.e. upregulation) of these genes in Rsp3+/- and other mutants. This was a very restricted analysis (wing imaginal disc). Is this phenomenon observed in other discs/tissues that form adult structures?

We have thoroughly addressed this in the revision. Our new data shows that *GR64* mutants induce cell death also in leg and haltere discs, but not in eye discs (S2 Fig). We note however that the effect is strongest in wing discs.

Reviewer #3 (Public Review):

I found this paper quite satisfying, because it is an excellent example of "following the science" to discover something new and totally unexpected about the way cells work. I thought the story was compelling, the presentation was clear and well written, and that the impact will be significant.

The authors followed up on observations made in a previous paper (Kucinski et al., 2017), where they compared gene expression in wild type flies versus flies heterozygous for a mutation in a ribosomal protein gene or another gene, mahjong, associated with proteotoxic stress. The analysis resulted in a long list of differentially expressed genes, some of which they explored in that paper. Here, they explore the reasons for the unusual upregulation of a family of gustatory receptors. Rather than representing a meaningless case of gene misregulation, they found that increased expression of these receptors is an adaptive response to proteotoxic stress. In this way, they implicated the receptors in regulating pathways that no one had suspected them of regulating.

I have no criticisms of the authors' choice of experiments, the methods they used, their interpretations or their presentation. The major strength of this paper is the soundness of the methods and results. It is an exceptionally "clean" and well-constructed report.

For me, the significance of the work relates to the long history of ribosomal protein gene haploinsufficiency in Drosophila and the increasing importance of Drosophila to understanding cellular responses to disrupted protein synthesis. A relatively short time ago, explanations for the Minute syndrome-the phenotypes seen in Drosophila when one copy of a ribosomal protein gene is eliminated-were speculative. The syndrome was considered an esoteric "fly thing". Over the past decade or so, the phenotypes have been tied to an assortment of cell stresses. A major challenge now is sorting through all the ways ribosome dysfunction affects cellular pathways. It is a complicated situation, but it mirrors cellular defects in human ribosomopathies and other diseases affecting protein synthesis. This paper is an excellent example of the relevance, importance and productivity of such studies: a new cellular pathway linking gustatory receptors to cellular stress responses has now been discovered from its contribution to the Minute syndrome.

We would like to thank reviewer #3 for their supportive words of the project and of our findings.

Reviewer #3 (Recommendations for the authors):

After reading this paper, one is left with the impression that what you describe for reduce RpS3 dosage is universal to reduced dosage for all Rp genes. I would like to have seen some statement that interactions between RpS3 and the Gr64 genes might be RpS3 specific. Better yet, I would like to have seen at least some experimental evidence showing the same interactions with another Rp gene. While I understand why you think the effects are probably not RpS3 specific, I urge to be hesitant and modest in generalizing. I recommend including appropriate caveats in the text.

This is indeed a very important point, which we have thoroughly addressed in the revision. We show that mutations in Gr64 induce an increase in cell death in two additional *Rp* mutants (S1 Fig). Our new data shows that *GR64* mutants induce cell death also in leg and haltere discs, but not in eye discs (S2 Fig). We note however that the effect is strongest in wing discs.

The text is very well written, but I had a hard time understanding the section on "flux" beginning with line 156. "Flux" is not a word that is universally understood. I had to go back to your previous paper to figure out what you were talking about and to understand the purposes of your ProteoFlux and ReFlux tools. For ease of reading, I strongly recommend that you use more familiar phrases such as "import and degradation", "elimination", etc. and provide better descriptions of the ProteoFlux and ReFlux tools.

Thanks for pointing this out. We have improved terminology and replaced the term flux with more accessible terms for ease of reading.

Because an asterisk is used to denote an unknown allele, I wanted to know why you used RpS3[*] in the allele designation in the experiments associated with Figure 1, but I could find no description of the origin of the allele. From context, I suspect it came from Bloomington stock 5699. Please include a note about its origin somewhere (or, better yet, sequence it and the known alleles and identify it definitively).

Thanks for pointing this out. Indeed, it refers to Bloomington stock 5699, and this is now explained in the text.

On line 66, you meant to use "proximate", not "proximal".

Thanks.

You should use FlyBase nomenclature for the genetic elements in your experiments, especially transgenes, at least once so that readers will not have to guess the correspondences between your lab notations and the carefully curated FlyBase entries describing them. Shortened symbols are OK for the text, but standardized symbols should be included in the Materials and Methods. This should be done for the sake of accuracy and reproducibility and to show that you value the efforts of the people who work hard organizing and archiving genetic information for you. Along the same lines, you should acknowledge research resources such as FlyBase that have enabled your work by explicitly citing their grants.

Thanks for pointing this out. We have improved referencing and use of FlyBase nomenclature throughout.
